# Impact of celiac disease on patients with familial Mediterranean fever: a nationwide study based on the Türkiye database

**DOI:** 10.3389/fimmu.2025.1675131

**Published:** 2025-12-10

**Authors:** Rıfat Bozkuş, Serdar Can Güven, Salih Başer, Nuray Yılmaz Çakmak, Hasan Satış, Emin Gemcioğlu, Naim Ata, Şuayip Birinci

**Affiliations:** 1 Department of Internal Medicine, Ankara Etlik City Hospital, Ankara, Türkiye; 2 Department of Rheumatology, Ankara Bilkent City Hospital, Ankara, Türkiye; 3 Department of Internal Medicine, Ankara Bilkent City Hospital, Ankara, Türkiye; 4 Department of Rheumatology, Dr. Abdurrahman Yurtaslan Ankara Oncology Training and Research Hospital, Ankara, Türkiye; 5 Republic of Türkiye Ministry of Health Strategy Development Department, Ankara, Türkiye; 6 Ministry of Health of the Republic of Türkiye, Ankara, Türkiye

**Keywords:** autoinflammatory disorders, autoimmunity, colchicine resistance, comorbidity, familial Mediterranean fever, celiac disease

## Abstract

**Introduction:**

Familial Mediterranean fever (FMF) and celiac disease (CD) are both immune-mediated disorders that may share overlapping inflammatory mechanisms. This study aimed to evaluate the clinical features, comorbidities, inflammatory markers, and treatment regimens in adult FMF patients with and without concurrent CD.

**Methods:**

In this retrospective, multicenter, nationwide study, data were obtained from the Turkish Ministry of Health’s National Electronic Database (e-Nabız). Diagnoses of FMF and CD were determined using ICD-10 codes and validated through clinical, serological, and histopathological records. A control group of FMF patients without CD was matched by age and sex. Demographic characteristics, comorbid conditions, medication use, emergency visits, surgical history, and serum amyloid A levels were analyzed.

**Results:**

Out of 184,786 adult FMF patients, 523 had coexisting CD and were compared with 523 matched FMF controls. Joint involvement rates were similar (p = 0.063), while comorbidities such as thyroid dysfunction, diabetes, depression (all p < 0.001), migraine (p = 0.009), and fibromyalgia (p= 0.003) were more prevalent in FMF–CD patients. Emergency visits for abdominal pain (p < 0.001) and peritonitis (p = 0.005) were significantly higher. Use of colchicine (compressed form), methotrexate, hydroxychloroquine, and methylprednisolone was also elevated (all p < 0.05).

**Conclusion:**

Coexisting CD in FMF patients is associated with a higher comorbidity burden, increased indirect inflammation, and greater use of immunomodulatory drugs. These findings suggest a more complex clinical profile but are limited by the absence of standardized disease activity metrics. Prospective studies using validated indices are needed for clearer insight and tailored management.

## 1 Introduction

Familial Mediterranean fever (FMF) is an autosomal recessive autoinflammatory disorder characterized by recurrent episodes of fever, abdominal pain, and serositis. It predominantly affects individuals of Eastern Mediterranean origin and is caused by mutations in the MEFV gene, resulting in excessive interleukin-1β (IL-1β) production and systemic inflammation. The clinical picture of the disease is heterogeneous; while some patients experience mild, infrequent attacks, others experience more severe, frequently recurring attacks, impairing quality of life and increasing the risk of long-term organ damage. If left untreated, FMF can lead to serious complications, such as secondary amyloidosis and renal failure. Colchicine remains the cornerstone of treatment. Colchicine resistance or intolerance, seen in approximately 5-10% of patients, remains a significant therapeutic challenge and may require biologic therapy such as IL-1 inhibitors or combination regimens. Furthermore, lifelong treatment and follow-up are essential to prevent complications such as amyloidosis, but adherence to treatment can be challenging due to lifelong medication use and side effects. Due to its chronic relapsing course and high prevalence in countries like Turkey, FMF is not only a clinical but also a public health problem, requiring early diagnosis, regular follow-up, and multidisciplinary monitoring (1, 2).

Based on data from our national study cohort derived from the Turkish national health database (e-Nabız), 239,175 patients with FMF were identified, including 184,786 adults. These numbers indicate a substantial national disease burden, while underreporting and undiagnosed cases suggest that the actual prevalence is likely higher (3).

Celiac disease (CD) is a chronic, immune-mediated enteropathy caused by an inappropriate immune response to dietary gluten in genetically predisposed individuals, particularly those carrying the HLA-DQ2 or HLA-DQ8 alleles. Although the disease affects approximately 1% of the population worldwide, a significant proportion of cases remain undiagnosed, representing a significant public health problem. CD presents a wide clinical spectrum, ranging from classic gastrointestinal findings such as diarrhea, malabsorption, and abdominal pain to extraintestinal manifestations such as anemia, osteoporosis, infertility, and neurological symptoms. Diagnosis is based on serologic testing and confirmation by duodenal biopsy. Disease severity and histopathologic findings range from mild mucosal inflammation to total villous atrophy according to the Marsh-Oberhuber classification. Persistent inflammation and nutrient malabsorption can lead to long-term complications, and CD is frequently associated with autoimmune comorbidities, particularly autoimmune thyroiditis and type 1 diabetes. Lifelong adherence to a strict gluten-free diet remains the cornerstone of treatment; however, maintaining adherence is often difficult due to social, economic, and psychological factors. Furthermore, CD potential for multisystem involvement, its association with nutritional deficiencies and autoimmune disorders, and the need for early diagnosis, patient education, and long-term multidisciplinary follow-up remain a significant clinical and public health problem (4–6).

FMF and CD have overlapping clinical features, including abdominal pain, diarrhea, joint pain, and arthritis. Both diseases are associated with a number of autoimmune and inflammatory comorbidities, including thyroid disorders, inflammatory arthritis, and connective tissue diseases. For these reasons, the coexistence of FMF and CD can potentially complicate differential diagnosis because symptoms of one disorder can mask or mimic those of the other. Furthermore, both conditions involve chronic inflammation and immune dysregulation, which may contribute to shared pathogenic pathways and mutual disease exacerbation. Despite these similarities and commonalities, the potential relationship between FMF and CD remains poorly understood, and most previous studies have been limited to small sample sizes or pediatric cohorts. This highlights the need for large-scale, adult-based analyses to better understand the frequency and clinical and therapeutic implications of comorbidities in the coexistence of these two diseases (7–10).

Previous studies investigating this relationship have been limited by small sample sizes and have predominantly focused on pediatric populations, resulting in inconsistent findings and leaving significant gaps regarding adult patients (8–10). To our knowledge, there are no previous large-scale studies addressing this association specifically in adults, making the present study the first nationwide analysis to explore the clinical impact of celiac disease in adult FMF patients. Given the overlapping symptomatology and shared immunopathogenic mechanisms of both disorders, the current study aims to assess the coexistence and clinical impact of CD in adult FMF patients using a large-scale, national dataset from Türkiye. Specifically, we examine whether the presence of CD is associated with increased attack frequency, abdominal pain, disease severity, and treatment resistance—particularly a reduced response to colchicine. We hypothesize that comorbid CD may exacerbate the clinical burden of FMF by amplifying systemic inflammation, worsening symptom severity, and diminishing treatment efficacy.

## 2 Materials and methods

### 2.1 Study design

This retrospective, observational, nationwide cohort study utilized data from the e-Nabız, under official supervision. Established in 2015, *e-Nabız* is a comprehensive health information system accessible only to authorized individuals and institutions. It contains clinical data from over 80 million individuals residing in Türkiye, including demographics, ICD codes, laboratory results, medication histories, and comorbidities. Integrated with the National Healthcare Information System (NHIS), *e-Nabız* has provided patient data since January 1, 2016, using big data Technologies (3). The study was conducted in accordance with the Declaration of Helsinki and approved by the Turkish Ministry of Health, with a waiver of informed consent granted for retrospective data analysis (approval number: 95741342-020).

### 2.2 Identification of patients and cohort selection

A nationwide FMF cohort was created by identifying adult patients (≥18 years of age) who had an ICD-10 code (E85.0) for FMF recorded at least twice, at least three months apart, between January 1, 2017, and December 31, 2023. Within this cohort, CD patients were identified using an ICD-10 code (K90.1) recorded at least three separate times. Diagnosis was confirmed by a physician’s report indicating the need for a gluten-free diet and at least one positive serological marker (anti-gliadin IgA/IgG, anti-transglutaminase IgA/IgG, or anti-endomysium antibodies). In addition, upper gastrointestinal endoscopy and histopathological reports were reviewed. Patients with biopsy-confirmed intraepithelial lymphocytosis, crypt hyperplasia, and/or villous atrophy were considered histologically confirmed CD cases. Patients lacking histopathological confirmation were not included. Control patients had no ICD codes, laboratory results, pathology results, or endoscopic findings suggestive of CD; they were not subjected to additional screening. Information on adherence to a gluten-free diet was not available in the national registry and could not be analyzed. A control group of 523 FMF patients without CD or other autoimmune/rheumatological disorders was randomly selected and matched for age and sex.

All eligible adult FMF patients (n = 523) with a confirmed concurrent diagnosis of CD in the national database were included as the case group. To ensure statistical comparability, an equal number of FMF patients without CD (n = 523) were selected as the control group using a 1:1 matching ratio. Frequency matching by age and sex was applied to ensure that the demographic distribution of the control group mirrored that of the case group. This approach preserved the true female predominance observed in the case group (74.4% of women and 25.6% of men) rather than imposing an artificial 1:1 sex ratio, because the primary objective of the study was to compare general clinical characteristics between the groups rather than investigate sex-specific differences. This matching strategy allowed for balanced group sizes, minimized selection bias, and maintained representativeness of the real-world population included in the national database.

### 2.3 Collected variables

Data were extracted for all eligible patients from the e-Nabız. The following variables were obtained for subsequent analyses: demographic characteristics—patient age (expressed as median and range), sex (male/female), and all-cause mortality.

Clinical features and disease-related outcomes: for FMF patients, presence of joint involvement, number of emergency department (ED) visits related to FMF attacks (median and range), documented history of abdominal surgery, and previous hospital admissions with diagnostic codes for peritonitis or acute appendicitis were recorded. For CD diagnosis, positivity of anti-gliadin IgA/IgG, anti-transglutaminase IgA/IgG, and presence of duodenal biopsy findings including intraepithelial lymphocytosis, crypt hyperplasia, and villous atrophy were evaluated. Histological grading was recorded, when available, according to the modified Marsh classification.

FMF disease severity was assessed using indirect clinical indicators, including elevated serum amyloid A (SAA) levels, increased frequency of emergency department visits related to FMF attacks, incidence of peritonitis, history of abdominal surgery, and types of medications administered. These surrogate markers were utilized given the retrospective design of the study and the absence of standardized disease activity scores within the database.

Information on prescribed medications was extracted, including colchicine (both standard and compressed formulations). The standard formulation refers to the conventional colchicine tablets available in Türkiye, while the compressed formulation (commonly sourced from France) represents a coated, film-compressed version with altered pharmacokinetic properties, including potentially slower dissolution and modified gastrointestinal tolerability, which may influence patient adherence and tolerability profiles. Other medications included disease-modifying antirheumatic drugs (DMARDs) such as methotrexate, sulfasalazine, and hydroxychloroquine; corticosteroids including methylprednisolone and deflazacort; and biologic agents comprising IL-1 inhibitors (anakinra, canakinumab), TNF-α inhibitors (adalimumab, etanercept, infliximab, golimumab, certolizumab pegol), an IL-6 inhibitor (tocilizumab), an IL-17 inhibitor (secukinumab), an IL-12/23 inhibitor (ustekinumab), and Janus kinase (JAK) inhibitors (baricitinib, tofacitinib).

Comorbid conditions were identified using ICD-10 codes and included diabetes mellitus, hypertension, autoimmune thyroid disorders, depression, fibromyalgia, pernicious anemia, migraine, and alopecia areata.

All variables were obtained through structured queries using diagnostic codes, laboratory result registries, procedure reports, and medication prescription records within the national health information system. Comparative analyses were conducted between FMF patients with and without celiac disease.

### 2.4 Statistical analysis

All statistical analyses were conducted using IBM SPSS Statistics for Windows, Version 21.0 (IBM Corp., Armonk, NY, USA). Continuous variables were assessed for normality using the Kolmogorov–Smirnov test. Variables not exhibiting normal distribution were expressed as median (minimum–maximum) and compared between groups using the Mann–Whitney U test. Categorical variables were presented as absolute numbers and percentages, and intergroup comparisons were performed using the Pearson’s Chi-square test or Fisher’s Exact test, depending on expected cell counts. A two-tailed p-value < 0.05 was considered statistically significant.

## 3 Results

A total of 239,175 patients with an FMF diagnosis were identified in the analysis. Of these, 184,786 individuals over the age of 18 were selected. Patients with inflammatory bowel disease, other rheumatological diseases, and immunodeficiency were excluded from the study, and a cohort of 523 patients with both FMF and CD was identified. The control group consisted of 523 age- and sex-matched patients without additional rheumatological diseases and followed only for FMF. Autoantibody positivity and pathological grades of CD patients are presented in **Supplementary Table S1**.

The mean age of patients in both groups was 38 years (range: 18–63), and 389 (74.4%) were female (*p* = 1.000). Joint involvement was detected in 266 (50.90%) patients in the FMF+CD group and in 236 (45.10%) patients in the control group, with no statistically significant difference between them (*p* = 0.063). Regarding thyroid diseases, thyroid dysfunction was observed in 128 (24.50%) patients in the case group and 72 (13.80%) in the control group, with a statistically significant difference (*p* = 0.001). When focusing on autoimmune thyroid disease specifically, autoimmune thyroiditis was found in 31 (5.93%) patients in the case group and 14 (2.68%) in the control group (*p* = 0.01). Among other comorbidities, diabetes mellitus (22.37% *vs*. 12.62%, *p* < 0.001), depression (62.33% *vs*. 45.51%, *p* < 0.001), migraine (16.63% *vs*. 11.09%, *p* = 0.009), and fibromyalgia (23.71% *vs*. 16.44%, *p* = 0.003) were significantly more prevalent in FMF+CD patients compared to the control group. No significant differences were observed for other comorbidities, including hypertension, pernicious anemia, hyperlipidemia, alopecia areata, and malignancy (**Table 1**).

**Table 1:** Demographic and clinical data of patients and distribution of comorbidities.

Variable	FMF with CD (case group)	FMF only (control group)	All patients	p*
Age, median (min-max)	38 (18-83)	38 (18-83)	38 (18-83)	1,000
Gender, Female, n (%)	389 (74,38)	389 (74,38)	778 (74,38)	1,000
Mortality, n (%)	12 (2,29)	8 (1,53)	20 (1,91)	0,366
Joint Involvement, n (%)	266 (50,86)	236 (45,12)	502 (47,99)	0,063
Thyroid Dysfunction, n (%)	128 (24,47)	72 (13,77)	200 (19,12)	0,000
Autoimmune Thyroid, n (%)	31 (5,93)	14 (2,68)	45 (4,30)	0,010
Diabetes Mellitus, n (%)	117 (22,37)	66 (12,62)	183 (17,50)	<0,001
Depression, n (%)	326 (62,33)	238 (45,51)	564 (53,92)	<0,001
Pernicious Anemia, n (%)	14 (2,68)	21 (4,02)	35 (3,35)	0,229
Fibromyalgia, n (%)	124 (23,71)	86 (16,44)	210 (20,08)	0,003
Alopecia Areata, n (%)	7 (1,34)	5 (0,96)	12 (1,15)	0,561
Migraine, n (%)	87 (16,63)	58 (11,09)	145 (13,86)	0,009

FMF, Familial Mediterranean fever; CD, celiac disease; *p value between FMF and CD *vs* only FMF group.

When the number of emergency department admissions due to abdominal pain was analyzed, the case group had a mean of 31 admissions (range: 0–346), compared to 21 admissions (range: 0–246) in the control group, which was statistically significant (*p* = 0.001). Emergency or hospital admissions due to peritonitis were observed in 20 (3.80%) patients in the case group and 6 (1.10%) in the control group (*p* = 0.005). No significant differences were found between the groups regarding acute appendicitis (*p* = 0.539) or emergency abdominal surgery (*p* = 0.111) (**Table 2**).

**Table 2:** Number of patients applying to the emergency department and clinical features.

Variable	FMF with CD (case group) (n=523)	FMF only (control group) (n=523)	All patients (n=1046)	p*
Emergency department visit count, median (min-max)	31 (0-346)	21 (0-246)	25 (0-346)	0,000
Ever abdominal surgery procedure count, n (%)	21 (4,02)	12 (2,29)	33 (3,15)	0,111
Ever admission with a diagnosis of peritonitis, n (%)	20 (3,82)	6 (1,15)	26 (2,49)	0,005
Ever admission with a diagnosis of acute appendicitis, n (%)	38 (7,27)	33 (6,31)	71 (6,79)	0,539

FMF, Familial Mediterranean fever; CD, celiac disease; *p value between FMF and CD *vs* only FMF group.

The serum amyloid A positivity rate at any time was significantly higher in patients with both FMF and CD compared to those with FMF alone (72.12% *vs*. 59.76%, *p* = 0.039) (**Table 3**).

**Table 3:** Comparison of serum amyloid A levels of patients.

Serum amyloid A status		FMF with CD (case group)		FMF (control group)		All patients		p*
		n	%	n	%	n	%	
Ever measured serum amyloid A	Normal	29	27,88	66	40,24	95	35,45	0,039
	Elevated	75	72,12	98	59,76	173	64,55	

FMF, Familial Mediterranean fever; CD, celiac disease; *p value between FMF and CD vs only FMF group.

Regarding medical treatment, colchicine use was similar between the two groups (*p* = 0.260). However, the use of compressed colchicine was significantly more common in the FMF+CD group compared to controls (*p* = 0.001). Among other drugs used in FMF treatment, methotrexate (*p* = 0.003), hydroxychloroquine (*p* = 0.020), and methylprednisolone (*p* = 0.001) were significantly more frequently prescribed in the FMF+CD group. A borderline significance was observed for anakinra use in the case group (*p* = 0.057) (**Table 4**).

**Table 4:** Distribution of drug treatments used by patients.

Drug class	Drug name	FMF + CD (case group), n (%)	FMF Only (control group), n (%)	Total, n (%)	p*
Colchicine formulations	Standard colchicine	475 (90.82)	485 (92.73)	960 (91.78)	0.260
	Compressed colchicine	45 (9.37)	19 (3.63)	64 (6.12)	0.001
Corticosteroids	Methylprednisolone	147 (28.11)	99 (18.93)	246 (23.52)	<0.001
	Deflazacort	7 (1.34)	6 (1.15)	13 (1.24)	0.780
Conventional DMARDs	Methotrexate	39 (7.46)	17 (3.25)	56 (5.35)	0.003
	Sulfasalazine	37 (7.07)	39 (7.46)	76 (7.27)	0.812
	Hydroxychloroquine	45 (8.60)	26 (4.97)	71 (6.79)	0.020
Biologics – IL-1 inhibitors	Anakinra	8 (1.53)	2 (0.38)	10 (0.96)	0.057
	Canakinumab	11 (2.10)	5 (0.96)	16 (1.53)	0.131
Biologics – TNF inhibitors	Adalimumab	8 (1.53)	8 (1.53)	16 (1.53)	1.000
	Etanercept	8 (1.53)	3 (0.57)	11 (1.05)	0.130
	Infliximab	11 (2.10)	5 (0.96)	16 (1.53)	0.131
	Golimumab	3 (0.57)	1 (0.19)	4 (0.38)	0.312
	Certolizumab pegol	3 (0.57)	4 (0.76)	7 (0.67)	0.500
Other biologics	Secukinumab (IL-17 inh.)	2 (0.38)	3 (0.57)	5 (0.48)	0.500
	Ustekinumab (IL-12/23 inh.)	1 (0.19)	0 (0.00)	1 (0.10)	0.500
	Tocilizumab (IL-6 inh.)	5 (0.96)	2 (0.38)	7 (0.67)	0.226
JAK inhibitors	Tofacitinib	3 (0.57)	0 (0.00)	3 (0.29)	0.125
	Baricitinib	1 (0.19)	1 (0.19)	2 (0.19)	0.750

FMF, Familial Mediterranean fever; CD, celiac disease; * p value between FMF and CD *vs* only FMF group.

Note: Drugs are grouped by pharmacological class. Colchicine formulations are shown separately, and DMARDs, corticosteroids, biologics, and JAK inhibitors are indicated with subheadings.

## 4 Discussion

To our knowledge, this is the first study to evaluate adult patients with FMF in relation to the presence or absence of coexisting CD, with a specific focus on associated comorbidities, clinical course, and treatment characteristics. While several case reports and small-scale studies in pediatric populations have explored the relationship between FMF and CD, data on adult patients remain limited. FMF patients with concomitant CD demonstrated a higher prevalence of autoimmune thyroid disease, diabetes mellitus, depression, fibromyalgia, and migraine than those without CD. Additionally, the frequency of emergency department visits and reported peritonitis attacks was significantly higher in the FMF with CD group. These patients were also more frequently prescribed compressed colchicine regimens, and their mean SAA levels were elevated, indicating a potentially more severe or refractory disease phenotype in FMF patients with coexisting CD.

Familial Mediterranean fever and CD have overlapping clinical manifestations, particularly abdominal pain, diarrhea, and joint pain (11). Our study demonstrates important clinical implications of this disease association: patients with concurrent FMF and CD presented to the emergency department at a significantly higher rate for abdominal pain and had an increased incidence of peritonitis compared with controls, suggesting a synergistic inflammatory burden. The lack of increased surgical interventions (e.g., appendectomy) further supports the inflammatory, rather than structural, nature of these symptoms. These findings highlight the clinical importance of screening for CD in FMF patients presenting with persistent or recurrent abdominal symptoms, particularly given previous reports that comorbid autoimmune conditions may worsen the severity of FMF (12, 13).When these findings are evaluated together, they indirectly support our hypothesis that the coexistence of CD and FMF may increase the frequency of attacks and worsen disease activity.

The prevalence of diabetes mellitus, depression, migraine, fibromyalgia, and autoimmune thyroid disease was significantly higher in FMF patients with coexisting CD compared to those without CD. These findings align with previous research suggesting a shared susceptibility to autoimmune and inflammatory conditions in this patient population (14–16). For instance, the increased rate of diabetes mellitus may reflect the cumulative burden of chronic inflammation and genetic predisposition, both of which are implicated in FMF and CD. The higher prevalence of depression observed in the FMF–CD group is also consistent with literature reporting a link between chronic inflammation and mood disorders (17, 18). The national database used in this study does not systematically collect information regarding multidisciplinary follow-up or psychological counseling for patients with CD. Consequently, it was not possible to determine whether patients with CD and depression received structured psychosocial support, which is recommended in best-practice management guidelines. Similarly, the elevated frequency of migraine in this group may be explained by shared inflammatory pathways and altered gut–brain axis regulation, although the exact mechanisms remain to be elucidated (19).

Fibromyalgia, known to be associated with both autoinflammatory and autoimmune diseases, was also more common in patients with both FMF and CD. This may be related to overlapping inflammatory profiles, including elevated levels of IL-1β, IL-6, TNF-α, and IL-17, which are characteristic of FMF, CD, and fibromyalgia (20–22). Additionally, increased intestinal permeability (“leaky gut”), reported in both FMF and CD, may contribute to systemic immune activation and chronic pain syndromes, providing a possible mechanistic link to fibromyalgia (23, 24).

Autoimmune thyroid disorders were also significantly more common in the CD group with FMF. This may reflect HLA-associated genetic predispositions (e.g., HLA-DQ2/DQ8) as well as micronutrient deficiencies (e.g., iron, vitamin D, selenium, iodine) that can impair thyroid function and are common in CD (25–27). Although the relationship between FMF and thyroid dysfunction is less well established, pro-inflammatory cytokines such as IL-6 and TNF-α may play a role in thyroid dysregulation in FMF (28). When both diseases coexist, these mechanisms may combine and increase the risk of thyroid pathology, including non-autoimmune complications such as amyloidosis (29). Given the potential metabolic and cardiovascular effects of thyroid dysfunction, our findings suggest that regular thyroid function monitoring is important in FMF patients with concomitant CD.

These findings suggest that the coexistence of FMF and CD may be associated with a higher burden of comorbidities, particularly autoimmune and inflammatory conditions. This highlights the need for comprehensive, multidisciplinary clinical monitoring in this patient group to enable early detection and appropriate management of potential complications. In line with our initial hypothesis, this increased comorbidity burden may reflect amplified systemic inflammation and more complex disease behavior in patients with both FMF and CD.

FMF patients with coexisting CD had significantly higher rates of elevated SAA levels compared to those without CD. It is well known that SAA levels increase rapidly during FMF attacks and are widely accepted as markers of acute inflammation (30). Moreover, even outside of attack periods, low-grade elevations in SAA may indicate subclinical inflammation, which is particularly relevant for FMF due to its association with amyloidosis risk (31). The higher SAA levels observed in the FMF–CD group may reflect an enhanced inflammatory burden in these patients. Previous studies have emphasized that SAA is a highly sensitive biomarker for both clinical and subclinical inflammation in FMF (32). However, it should be noted that our study did not include direct measures of disease activity such as attack frequency, length of hospital stay, or the Autoinflammatory Disease Activity Index (AIDAI), a validated scoring system used to quantify disease activity in autoinflammatory conditions. Therefore, relying on surrogate indicators such as SAA levels and emergency department visits to infer disease severity represents a limitation. While these findings may suggest more pronounced inflammation in FMF patients with coexisting CD, further studies incorporating objective clinical activity measures are needed to confirm this association. Nonetheless, the higher SAA levels observed in the FMF–CD group provide additional indirect support for our hypothesis that coexisting CD may amplify systemic inflammation and contribute to greater disease activity in FMF.

While the overall use of colchicine was comparable between groups, the use of compressed colchicine was significantly more frequent among FMF patients with coexisting CD. In clinical practice, compressed colchicine is often preferred in cases of intolerance or suboptimal response to standard formulations. This observation may reflect increased colchicine resistance or intolerance in this subgroup; however, our dataset did not include direct information on treatment indications or response profiles, which limits interpretation. One possible explanation is that gastrointestinal symptoms or malabsorption related to CD may influence drug absorption and lead to perceived colchicine inefficacy. In line with this, previous studies have suggested that compressed colchicine formulations may offer improved pharmacokinetics, with more stable absorption and reduced gastrointestinal side effects—features that could be particularly advantageous in conditions like CD (33–36). Although our findings raise the possibility that FMF–CD coexistence may be associated with altered treatment needs or responses, causality cannot be established. These differences should be viewed as hypothesis-generating and warrant further investigation. In particular, future prospective studies incorporating standardized disease activity scores and pharmacokinetic analyses of colchicine formulations could clarify the clinical significance of these observations. Until such evidence is available, clinicians are advised to consider the possibility of underlying CD in FMF patients who exhibit signs of colchicine intolerance or resistance before initiating alternative therapies such as biologics. Individualized treatment planning and close monitoring remain essential in this complex patient population. The results suggest that immunomodulatory and anti-inflammatory agents—specifically methotrexate, hydroxychloroquine, and methylprednisolone—were prescribed significantly more frequently in FMF patients with coexisting CD than in those with FMF alone. However, the underlying reasons for medication choice (e.g., colchicine intolerance, autoimmune overlap, or disease severity) were not directly assessed in our dataset, which limits interpretation. Methotrexate has anti-inflammatory and immunomodulatory properties and is occasionally used in colchicine-resistant FMF, particularly when chronic arthritis is present (37). In the context of FMF–CD coexistence, shared inflammatory pathways—such as IL-1β-driven autoinflammation in FMF and T cell-mediated inflammation in the gut in CD—may contribute to a more complex immunological profile (2, 38). Based on this, the increased use of methotrexate could represent an effort to modulate overlapping inflammatory processes, though this interpretation remains hypothetical and requires further validation. Hydroxychloroquine, commonly used in autoimmune diseases, may also have been used as an alternative in cases with colchicine resistance or intolerance. While some case reports suggest its benefit in colchicine-resistant FMF or coexisting autoimmune conditions (39–41), there is currently no specific evidence supporting its routine use in FMF–CD overlap. Similarly, methylprednisolone—although not a first-line agent in FMF—is sometimes used short-term in colchicine-resistant cases, during acute inflammatory episodes, or in the presence of autoimmune comorbidities (42, 43). The more frequent use of these agents in the FMF–CD group may reflect a greater inflammatory burden or more complex treatment needs; however, direct measures of disease activity were not assessed in this study. Therefore, these findings should be interpreted cautiously and seen as hypothesis-generating. Further prospective studies, ideally incorporating standardized disease activity indices and treatment response criteria, are needed to better understand pharmacological management in this unique patient population.

Considering the role of specific cytokines in the pathogenesis of FMF, clinicians are increasingly turning to biologic agents targeting IL-1, IL-6, TNF-α, and JAK pathways when colchicine treatment is ineffective or intolerable^2^. The lack of a significant difference in biologic agent use between the groups may be attributed to the low number of patients receiving these treatments in both cohorts.

In Türkiye, national FMF treatment protocols recommend colchicine as first-line therapy for all patients, using standard or enteric-coated formulations, depending on tolerability and gastrointestinal side effects. In patients who do not respond adequately to maximally tolerated colchicine therapy or who develop complications such as amyloidosis, treatment is escalated to IL-1 inhibitors (such as anakinra or canakinumab), IL-6, TNF-α, and biologic agents targeting the JAK pathways, in accordance with national and international guidelines. In selected cases, particularly those with colchicine intolerance, frequent exacerbations, or incompletely controlled inflammatory features, additional therapies such as low-dose corticosteroids or conventional disease-modifying antirheumatic drugs (DMARDs) such as azathioprine and methotrexate, or sometimes biologics targeting other pathways, may be considered. These approaches are individualized based on disease severity, comorbidities, and response to previous therapies. The medication use patterns observed in this study are consistent with these treatment protocols.

These therapeutic differences between the CD with FMF and FMF-only groups indirectly support our initial hypothesis that concurrent CD may be associated with increased disease activity and altered treatment responses, and call for more complex therapeutic strategies.

This study has several limitations. First, it has a retrospective and cross-sectional design, and the data were obtained through ICD-10 codes, which limits the ability to establish causal relationships or perform precise prevalence calculations between FMF and CD. Another limitation involves data integration. Combining large-scale data from different sources requires standardized formatting, which may not always be feasible and may introduce inconsistencies. Although this is a common issue in nationwide health database studies, the large sample size can help mitigate potential errors. While our findings suggest an increased inflammatory disease burden in patients with coexisting FMF and CD, direct measures of disease activity—such as attack frequency, length of hospitalization, or standardized activity scores like AIDAI—were not available. Instead, we relied on surrogate markers including the frequency of emergency department visits, SAA levels, the occurrence of peritonitis, the presence of comorbid conditions, and differences in treatment regimens. This assessment, based on indirect indicators of disease severity, represents a limitation that must be recognized when interpreting the results. Moreover, the study design is only suited to evaluating certain clinical features and treatment patterns in FMF and CD patients and does not represent a comprehensive epidemiological assessment. Therefore, the generalizability of our findings to broader clinical practice should be approached with caution, and further prospective studies incorporating standardized disease activity scores are needed to confirm and expand upon our results. Another limitation of this study is the lack of information in the national database regarding psychological support, nutritional counseling, and multidisciplinary follow-up practices for patients. These factors may influence treatment adherence and the clinical presentation of the disease.

## 5 Conclusion

This large-scale, real-world study utilizing the Turkish national health database highlights notable differences in clinical features and treatment patterns among FMF patients with coexisting CD. The higher rates of comorbidity, increased inflammatory marker levels, and differences in treatment modalities in patients with both conditions support our hypothesis that the coexistence of the two conditions increases disease severity and decreases treatment response. While these findings suggest a possible link between CD and heightened FMF disease activity, the absence of standardized disease activity scores limits definitive conclusions. Future prospective studies incorporating clinical activity indices, validated severity scores, and pharmacokinetic assessments of colchicine formulations are warranted to better understand the underlying mechanisms and to optimize management strategies for this unique patient subgroup.

## Data Availability

The original contributions presented in the study are included in the article/**Supplementary Material**. Further inquiries can be directed to the corresponding author.
