# Supplementary material for: Impact of celiac disease on patients with familial Mediterranean fever: a nationwide study based on the Türkiye database
Source: Front Immunol. 2025 Dec 10;16:1675131. doi: 10.3389/fimmu.2025.1675131 (PMC12731243; doi:10.3389/fimmu.2025.1675131)
Supplement: Supplementary file 1 [file Table1.docx]

**Supplementary Table 1. Autoantibody positivities and histopathological gradings of celiac disease patients**

| Test / Grading | No Result¹, n (%) | Negative, n (%) | Positive, n (%) |
| --- | --- | --- | --- |
| Autoantibodies |  | | |
| Anti-gliadin IgG | 229 (43.79) | 201 (38.43) | 93 (17.78) |
| Anti-gliadin IgA | 179 (34.23) | 192 (36.71) | 152 (29.06) |
| Tissue transglutaminase IgA | 217 (41.49) | 206 (39.39) | 100 (19.12) |
| Tissue transglutaminase IgG | 285 (54.49) | 202 (38.62) | 36 (6.88) |
| Histopathological grading² |  | | |
| No result¹ | 259 (49.52) |  | |
| Marsh 0 | 7 (1.34) |  |  |
| Marsh 1 | 82 (15.68) |  |  |
| Marsh 2 | 59 (11.28) |  |  |
| Marsh 3 (overall) | 97 (18.54) |  |  |
| 3a | 1 (0.19) |  |  |
| 3b | 3 (0.57) |  |  |
| 3c | 8 (1.53) |  |  |

¹ “No result” indicates that the corresponding laboratory or pathology report was not retrievable from the national database, either because the data entry was incomplete or the result was not available at the time of extraction. However, this does not imply absence of diagnostic evaluation, as some records may have been performed but not digitally documented in the registry.

² Histopathological grading was performed using the modified Marsh classification: Marsh 0 (normal mucosa), Marsh 1 (intraepithelial lymphocytosis), Marsh 2 (crypt hyperplasia), Marsh 3a–c (progressive degrees of villous atrophy — 3a: partial, 3b: subtotal, 3c: total villous atrophy).
